# Mid-treatment Axillary Lymph Node Metastasis of Undifferentiated Nasopharyngeal Carcinoma: An Unprecedented Case

**DOI:** 10.7759/cureus.116004

**Published:** 2026-09-09

**Authors:** Btissam Amrani, Soufiane Berhili, Lamyae Jabri, Ahmed BenSghier, Mohamed Moukhlissi, Loubna Mezouar

**Affiliations:** 1 Radiation Oncology, Faculty of Medicine and Pharmacy of Oujda, Mohammed First University, Oujda, MAR; 2 Radiation Oncology, Centre Hospitalier Universitaire Mohammed VI, Oujda, MAR; 3 Radiotherapy, Centre Hospitalier Universitaire Mohammed VI, Oujda, MAR

**Keywords:** axillary lymph-node metastasis, concurrent chemoradiation therapy, nasopharyngeal carcinoma (npc), non-keratinizing squamous cell carcinoma, radiation & medical oncology

## Abstract

Nasopharyngeal carcinoma (NPC) usually spreads through the retropharyngeal and cervical lymph nodes, whereas axillary involvement is exceptionally rare and represents non-regional nodal disease. We report a 55-year-old man with cT2N3M0 undifferentiated non-keratinizing NPC (UCNT) who received induction gemcitabine-cisplatin chemotherapy followed by definitive concurrent chemoradiotherapy. After six radiotherapy fractions, the patient developed left axillary pain. Ultrasonography revealed ipsilateral axillary lymphadenopathy, and excisional biopsy demonstrated a poorly differentiated carcinoma morphologically compatible with the known nasopharyngeal primary. Following multidisciplinary review, concurrent chemoradiotherapy was completed as planned, and the patient was referred for consideration of systemic treatment. This case highlights the importance of promptly investigating newly developed axillary lymphadenopathy during treatment and obtaining histopathological confirmation to guide multidisciplinary management.

## Introduction

Nasopharyngeal carcinoma (NPC) is an epithelial malignancy that develops from the mucosal lining of the nasopharynx. Its global distribution is markedly uneven, with the highest incidence observed in East and Southeast Asia, intermediate incidence rates reported in North Africa (where our case was described), and substantially lower rates among predominantly White populations [1,2]. According to the World Health Organization classification, non-keratinizing carcinoma, either differentiated or undifferentiated, is the predominant histological subtype of NPC, particularly in endemic regions, and is strongly associated with Epstein-Barr virus infection [3].

Owing to the rich lymphatic network of the nasopharynx, cervical lymph node involvement is common at diagnosis and typically follows an orderly pattern, initially affecting the retropharyngeal and upper cervical nodal groups before extending to the lower neck. Poorly differentiated and undifferentiated subtypes exhibit a particularly marked predilection for lymphatic and distant spread, with the risk of systemic dissemination increasing according to the extent and distribution of nodal involvement. The most frequent sites of distant metastasis are the bones, lungs, and liver, with bone being the predominant site [4,5].

Axillary lymph node involvement falls outside the established regional lymphatic drainage pathways of the nasopharynx and is therefore classified as distant metastatic disease. Such involvement is exceptionally uncommon, with only a few cases documented in the literature. Most reported cases have occurred after completion of definitive treatment, typically in the setting of locoregional recurrence or widespread metastatic dissemination [6,7].

In this article, we describe a rare case of a 55-year-old man with NPC who developed an axillary lymph node metastasis shortly after the initiation of concurrent chemoradiotherapy following induction chemotherapy.

## Case presentation

A 55-year-old man was referred to our department for the management of an NPC. He had an eight-month history of a progressively enlarging left cervical mass associated with left-sided hearing impairment and recurrent episodes of epistaxis. These symptoms prompted an otorhinolaryngological evaluation and a nasopharyngeal biopsy. Histopathological examination established the diagnosis of undifferentiated non-keratinizing NPC.

Initial contrast-enhanced CT scan of the brain, neck, chest, abdomen, and pelvis demonstrated a nasopharyngeal tumor with parapharyngeal extension and multiple enlarged left cervical lymph nodes involving levels II, III, IVa, and V, without axillary lymph node involvement or distant visceral metastases. Bone scintigraphy showed no skeletal involvement. The disease was therefore staged as cT2N3M0 according to the eighth edition of the Union for International Cancer Control Tumor, Node, Metastasis (TNM) classification.

Following multidisciplinary discussion, the patient received three cycles of induction chemotherapy consisting of cisplatin 80 mg/m² on Day 1 and gemcitabine 1,000 mg/m² on Days 1 and 8 of each three-week cycle. He subsequently proceeded to definitive concurrent chemoradiotherapy. Intensity-modulated radiotherapy was delivered using a simultaneous integrated boost technique, with three dose levels administered over 35 fractions. The high-risk target volume encompassed the residual primary tumor and involved lymph nodes after induction chemotherapy. The intermediate-risk target volume included bilateral nodal levels Ib, II, III, IVa, Vb, VIIa, and VIIb, as well as the anatomical structures adjacent to the primary gross tumor volume considered to be at intermediate risk of microscopic involvement. The low-risk elective target volume encompassed bilateral nodal levels II, III, IVa, Va, Vb, VIIa, and VIIb. Total doses of 70 Gy (2 Gy /fraction), 63 Gy (1.8 Gy /fraction), and 56 Gy (1.6 Gy /fraction) were delivered to the high-, intermediate-, and low-risk target volumes, respectively, with concurrent weekly cisplatin at a dose of 35 mg/m².

After the sixth radiotherapy fraction, the patient began developing new-onset left axillary pain. Axillary ultrasonography revealed multiple enlarged left axillary lymph nodes, the largest measuring 26 × 17 mm. Given the unusual location of the lymphadenopathy and the need to establish its origin, an excisional biopsy of the largest lymph node was performed, without treatment interruption.

Histopathological examination demonstrated lymph node involvement by a poorly differentiated carcinoma morphologically compatible with the patient's known NPC. Immunohistochemical analysis showed focal CK5/6 positivity, whereas Epstein-Barr virus testing was negative (Figure 1).

**Figure 1:**
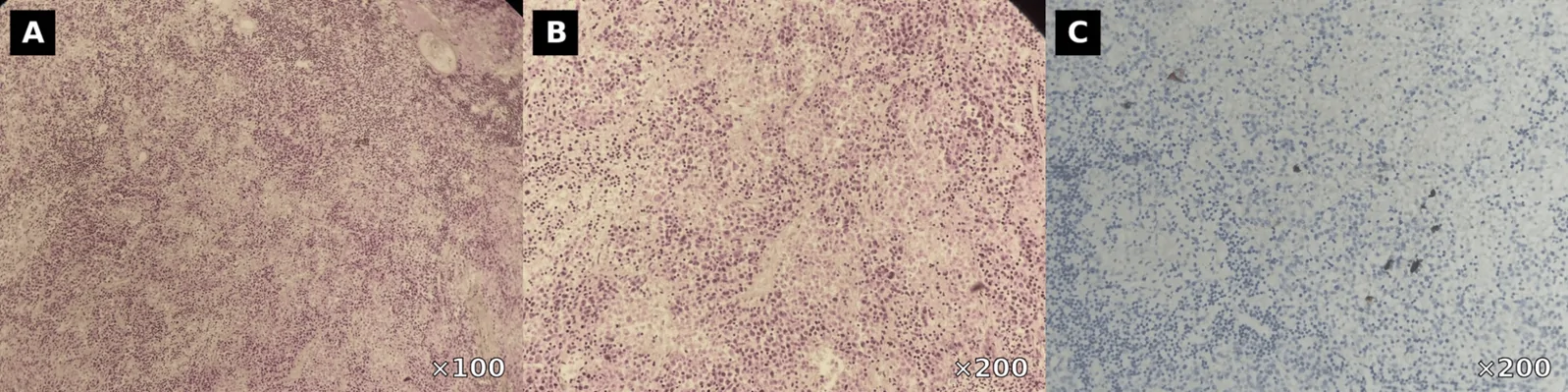
Histopathological findings of the excised left axillary lymph node. (A) Low-power hematoxylin and eosin-stained section showing lymph node infiltration by a poorly differentiated carcinomatous proliferation (×100). (B) Higher-power view showing large atypical tumor cells admixed with a prominent lymphoid infiltrate (hematoxylin and eosin; ×200). (C) Immunohistochemical staining for cytokeratin 5/6 showing focal expression in rare tumor cells (×200).

Taken together with the clinical history and morphological findings, these results supported the diagnosis of axillary lymph node metastasis from the NPC.

Subsequent contrast-enhanced computed tomography demonstrated an enlarged left axillary lymph node together with several smaller ipsilateral axillary nodes, supporting involvement of multiple left axillary nodes (Figure 2).

**Figure 2:**
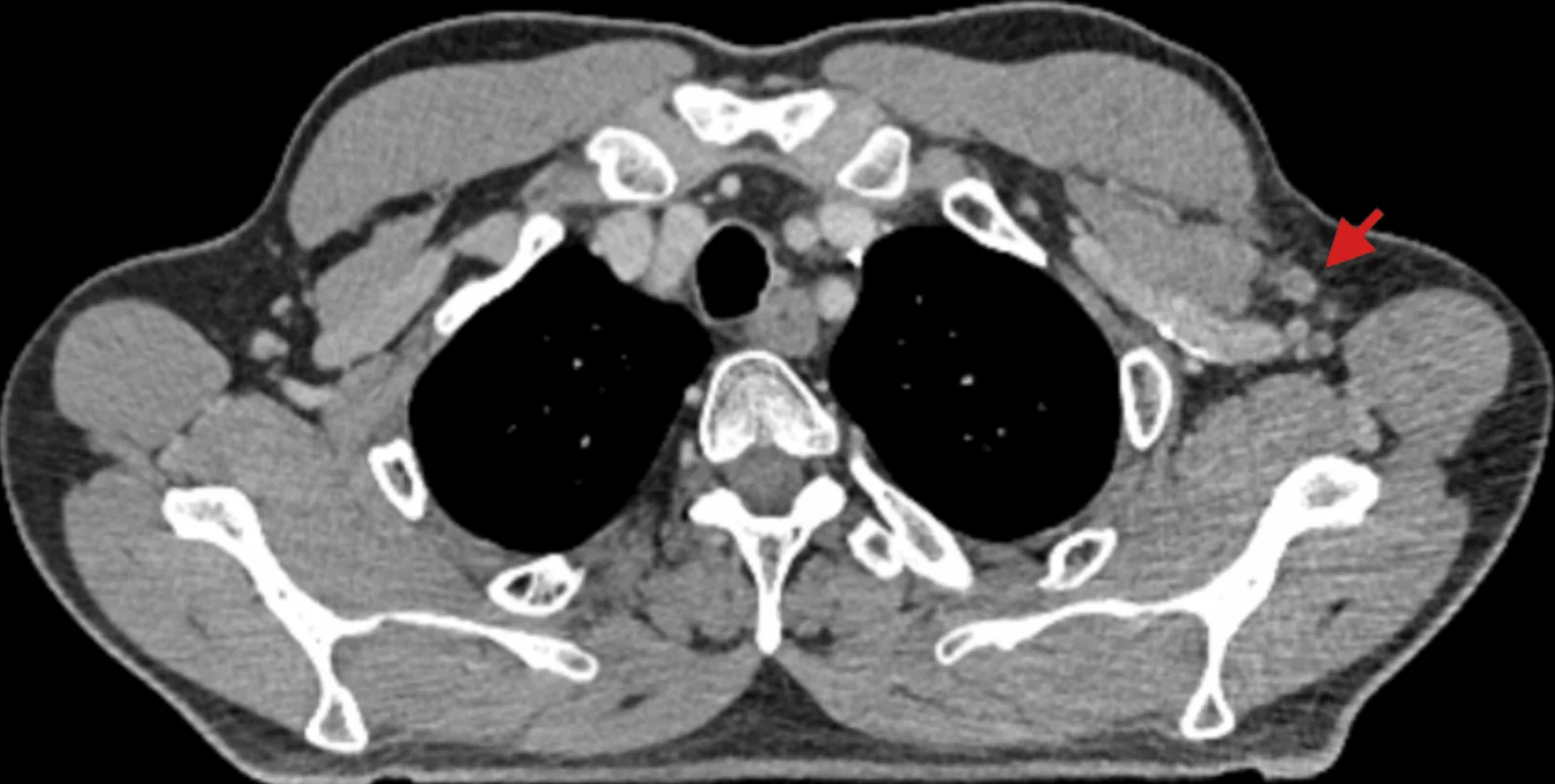
Axial contrast-enhanced computed tomography image showing an enlarged left axillary lymph node (red arrow).

Axillary nodal involvement was detected during the initial treatment course, with no other distant metastases identified on the available computed tomography imaging.

The case was discussed at a multidisciplinary oncology team meeting. Given that fewer than five axillary nodes were suspicious of malignancy, the disease was upstaged as oligometastatic. Since the axillary metastasis was detected during treatment, the decision was to continue the ongoing concurrent chemoradiotherapy as initially planned to preserve locoregional control of the bulky nasopharyngeal and cervical disease.

Following treatment completion, the patient was referred to the medical oncology department for consideration of systemic treatment of the metastatic disease. Subsequent response assessment with PET/CT was planned, after which the case would be rediscussed to determine further management.

## Discussion

NPC differs from most other head and neck carcinomas because of its marked propensity for early lymphatic dissemination, which usually follows a predictable and orderly pattern through the retropharyngeal and cervical lymph node chains [8]. The present case is notable for histologically confirmed left axillary lymph node involvement, on the same side as the initial cervical lymphadenopathy, detected during the initial course of treatment in a patient initially staged as cT2N3M0. Unlike most previously reported cases, in which axillary involvement occurred after completion of definitive treatment, often following a disease-free interval [7], the axillary lymphadenopathy in our patient became clinically apparent after induction chemotherapy and shortly after the initiation of concurrent chemoradiotherapy. This presentation illustrates an uncommon pattern of non-regional nodal spread and raises important questions regarding its timing, underlying mechanism, and therapeutic implications.

Regional nodal involvement in NPC generally follows a predictable and orderly pattern. A meta-analysis of 13 MRI-based studies comprising 2,920 patients found regional lymphadenopathy in approximately 85% of cases, with the retropharyngeal and level II lymph nodes representing the most frequently involved stations. Levels III, V, and IV were involved less frequently, while skip metastases were uncommon [4]. These findings reflect the usual lymphatic drainage of the nasopharynx toward the retropharyngeal and upper deep cervical chains, followed by inferior spread through the cervical nodal levels. In contrast, the axillary basin lies outside the regional lymphatic drainage territory of the nasopharynx. Accordingly, histologically confirmed axillary involvement attributable to NPC constitutes distant nodal metastasis (M1) rather than an extension of the cervical N category [9].

Axillary lymph node metastasis from NPC has been reported only sporadically, mainly in individual case reports and small case series. Most reported cases were metachronous, occurring months or even years after completion of definitive treatment [6,10-16]. Lee et al. described axillary involvement as a manifestation of disease relapse following chemoradiotherapy, whereas Kuo et al. reported an isolated axillary recurrence after a seven-year disease-free interval [6,12]. In other cases, axillary metastases occurred alongside cervical, supraclavicular, or mediastinal nodal recurrence [14,16]. Synchronous axillary involvement appears to be even less common. Yazici et al. reported axillary lymphadenopathy in a patient who also had bilateral cervical lymphadenopathy and bone metastasis [15]. Similarly, Hajra et al. described axillary involvement in the setting of extensive nodal and multiorgan metastatic disease [10]. A recent literature review further highlighted the exceptional nature of isolated axillary involvement and the considerable heterogeneity among reported cases regarding the timing of detection, disease burden, therapeutic management, and clinical outcomes [7].

The timing of axillary involvement in our patient cannot be established with certainty. Baseline conventional staging did not identify axillary or distant metastatic disease, whereas symptomatic axillary lymphadenopathy was detected after three cycles of induction chemotherapy and six radiotherapy fractions. Nevertheless, the available findings do not establish whether the axillary disease was already occult at baseline or emerged during treatment. Subsequent CT revealed another ipsilateral axillary lymph node, arguing against a truly solitary metastasis. Accordingly, this case represents a mid-treatment axillary lymph node metastasis detection, with no documented distant metastatic disease outside the axillary region.

The route of axillary spread from NPC remains uncertain. Proposed mechanisms include aberrant or retrograde lymphatic drainage caused by bulky lower cervical disease, lymphatic continuity between the lower neck and the axillary apex, and, less likely, hematogenous dissemination with secondary nodal colonization. Previous cervical surgery or radiotherapy may also alter lymphatic drainage and has been implicated in delayed axillary recurrence [7,12,17]. This mechanism appears less likely in our patient because axillary involvement was detected early during the initial treatment course. Conversely, the extensive ipsilateral cervical nodal burden, particularly at the lower cervical and level V stations, together with the ipsilateral axillary involvement, supports aberrant or retrograde lymphatic spread as a plausible explanation, although hematogenous dissemination cannot be excluded.

Given the unusual location of the lymphadenopathy in our case, tissue confirmation was essential. Excisional biopsy demonstrated a poorly differentiated carcinoma whose morphological features, interpreted in the context of the patient's known primary tumor, were consistent with metastatic NPC. Focal CK5/6 expression was compatible with squamous differentiation. Epstein-Barr virus-encoded small RNAs (EBER) in situ hybridization was negative in both the primary nasopharyngeal tumor and the axillary lymph node specimen. Overall, the concordant morphological, immunohistochemical, and EBER findings supported axillary metastasis from the known NPC.

The diagnosis consequently relied on the integration of morphology, immunohistochemical findings, clinical history, and imaging [7,15,18]. Comparison with the original nasopharyngeal biopsy is particularly valuable in this setting because unexpected axillary lymphadenopathy also raises alternative diagnoses, including lymphoma and primary malignancies of the breast, lung, skin, or upper limb [7,15].

Because no standard treatment has been established for axillary nodal metastasis from NPC, management must be individualized according to disease extent, previous treatment, and therapeutic intent [7]. In our patient, multidisciplinary review supported completion of the ongoing concurrent chemoradiotherapy and resumption of chemotherapy, followed by clinical and radiological reassessment. Given the absence of contemporaneous whole-body PET/CT and the subsequent detection of another ipsilateral axillary node, this approach should be regarded as an individualized decision rather than a standard therapeutic strategy, placing greater emphasis on the value of multidisciplinary discussion for managing complex cases such as ours.

## Conclusions

Axillary lymph node involvement represents a rare pattern of distant nodal spread in NPC, particularly when detected during the initial treatment course. This case emphasizes the need for careful evaluation of any new symptoms or lymphadenopathy arising outside the usual cervical drainage pathways, even during ongoing treatment. Prompt imaging, tissue confirmation, comprehensive restaging, and multidisciplinary reassessment are essential, as recognition of this unusual pattern may influence disease classification and subsequent therapeutic planning.
